# You must be myths-taken: Examining belief in falsehoods during the COVID-19 health crisis

**DOI:** 10.1371/journal.pone.0294471

**Published:** 2024-03-05

**Authors:** May Oo Lwin, Anita Sheldenkar, Pei Ling Tng

**Affiliations:** Wee Kim Wee School of Communication and Information, Nanyang Technological University, Singapore, Singapore; University of the Highlands and Islands, UNITED KINGDOM

## Abstract

The prevalence of health myths is increasing with the rise of Internet use. Left unaddressed, online falsehoods can lead to harmful behaviours. In times of crisis, such as the recent COVID-19 pandemic, the circulation of many myths is exacerbated, often to varying degrees among different cultures. Singapore is a multicultural hub in Asia with Western and Asian influences. Although several studies have examined health myths from a Western or Eastern perspective, little research has investigated online health falsehoods in a population that is culturally exposed to both. Furthermore, most studies examined myths cross-sectionally instead of capturing trends in myth prevalence over time, particularly during crisis situations. Given these literature gaps, we investigated popular myths surrounding the recent COVID-19 pandemic within the multicultural setting of Singapore, by examining its general population. We further examined changes in myth beliefs over the two-year period during the pandemic, and population demographic differences in myth beliefs. Using randomised sampling, two online surveys of nationally representative samples of adults (aged 21–70 years) residing in Singapore were conducted, the first between October 2020 and February 2021 (N = 949), and the second between March and April 2022 (N = 1084). Results showed that 12.7% to 57.5% of the population were unable to identify various myths, such as COVID-19 was manmade, and that three of these myths persisted significantly over time (increases ranging from 3.9% to 9.8%). However, belief in myths varied across population demographics, with ethnic minorities (Indians and Malays), females, young adults and those with lower education levels being more susceptible to myths than their counterparts (*p* < 0.05). Our findings suggest that current debunking efforts are insufficient to effectively counter misinformation beliefs during health crises. Instead, a post-COVID-19 landscape will require targeted approaches aimed at vulnerable population sub-groups, that also focus on the erroneous beliefs with long staying power.

## Introduction

Myths can be understood as widely circulated and believed ideas that are false and unsubstantiated by existing scientific facts [1]. They can range from statements that appear to sound logical or intuitive, to explanations based on flimsy or distorted evidence [1]. Health myths have existed for centuries. For instance, radioactive water was once a popular medicine touted to alleviate chronic pain in the 1900s [2], while claims that vaccines are harmful have been regularly promoted [3].

With global Internet penetration increasing to an all-time high [4], and the rising use of social media, there has been an alarming surge in the spread of health myths. Users can easily post falsehoods, either intentionally to cause harm, or out of misguided and uninformed concern, and these can be viewed by millions around the globe. In their systematic review, Wang and colleagues [5] found a sharp increase in the number of studies examining health misinformation from 2012 to 2018, particularly in the areas of infectious diseases and vaccines. Misinformation is expected to have increased exponentially since then with the COVID-19 pandemic [6].

While some misinformation beliefs might be innocuous, others can be injurious to health by causing harmful behaviours. For example, the myth in Northern Nigeria that people can develop cancer after exposure to mystical sources and that hospitals can exacerbate the symptoms may have prevented people from seeking appropriate and timely treatment [7]. Within certain communities in India, myths about menstruation being “dirty” have perpetuated the stigmatisation of menstruating individuals, leading to unhygienic practices that contribute to infections and mental health problems [8]. Over 20 years ago, Wakefield [9] published a later retracted and disproven paper claiming that childhood vaccinations lead to autism. This claim has driven parental hesitancy regarding child vaccinations and contributed to a resurgence in preventable childhood diseases [10]. A systematic review by Borges Do Nascimento and colleagues [11] found that misinformation can create more disharmony among people, lead to delays in obtaining healthcare, and contribute to misinterpretations of evidence. Health misinformation can also spur the use of potentially dangerous alternative medicines [12]. Left unchecked, the propagation of myths can lead to harmful behaviours that increase the health burden.

### Research objectives

Although there have been various studies examining health misinformation, myths can vary considerably between different cultures. Hence, this study aims to understand beliefs within a multicultural Asian context by examining three research questions. Firstly, to investigate popular health myths in Singapore’s multicultural setting during a health crisis, specifically the COVID-19 pandemic, we pose the question “What and how strong are beliefs surrounding prevalent COVID-19 myths among the Singapore public?” (RQ1). Secondly, most studies examining myths are cross-sectional. To understand the typology of myths which have long staying power, we further examine the question “How have beliefs in these COVID-19 myths shifted over time?” (RQ2). Finally, to understand factors associated with susceptibility to health misinformation, we examine the question “Are there demographic differences (age, gender and ethnicity) regarding these COVID-19 myth beliefs?” (RQ3). A better understanding of common myths would enable the creation of more targeted health communication strategies to tackle misinformation during current and future health crises.

### Background on COVID-19 myths

During times of crisis, such as public health emergencies, uncertainty and disruption to the normal routine can rapidly increase the spread of falsehoods [13, 14].

As a novel disease that first emerged in 2019, the COVID-19 pandemic rapidly evolved over time, with the resultant flows of emerging information causing great instability and anxiety among the general public. Enabled by a rise in Internet usage spurred by physical distancing regulations [15], and greater online health-seeking behaviours due to worries about in-person visits to doctors [16], myths about the disease have spread swiftly.

In early 2020, the World Health Organisation (WHO) classified the onslaught of COVID-19 misinformation as an “infodemic”, which they warned could lead to more uncertainty about appropriate behaviours and less trust in the authorities [17]. This infodemic has remained prevalent over the pandemic, with COVID-19 myths being spread through social media [18, 19]. For instance, there have been arson attacks on 5G phone masts in the United Kingdom after a conspiracy theory blamed 5G technology for spreading the virus [20], and poisoning incidents in Iran following the circulation of inaccurate statements that alcohol can treat COVID-19 [21]. The use of the anti-parasitic drug Ivermectin was promoted by a fringe group of doctors, contrary to official recommendations, and many who consumed it displayed adverse reactions [22]. Additionally, studies have found that those who believe in myths and conspiracy theories are less likely to comply with COVID-19 safety measures and less willing to get vaccinated [23, 24]. Hence, it is imperative to understand factors influencing beliefs in COVID-19 myths, to inform effective health communication strategies to stem the spread of false information.

The profile of COVID-19 myths may differ between various cultures, and what is prevalent in one country might not necessarily be generalisable to another. For instance, Alimardani and Elswah [25] highlighted how religious types of false information have become a particularly pronounced problem in the Middle East and North Africa region. In Ghana, the slow spread of the disease led many to initially believe that black genes conferred immunity to COVID-19 [26]. Elsewhere, in the United States, an early study showed that up to 43% of respondents believed that using antibiotics, hand dryers or mouthwash could prevent COVID-19 infection [27]. Therefore, it is useful to understand the specific myths present in different cultural contexts, which would allow governments to provide targeted health communication.

Singapore is a multicultural hub in Asia that draws cultural influences from China, Southeast Asia and India. The population consists of 74% Chinese, 14% Malays, 9% Indian, and 3% Other ethnic groups, with a median age of 41.5 years [28]. With English being the most spoken language for almost half of the population [29], the country also gets exposure to Western media. It has been ranked the number one smart city in the world [30], with an Internet and social media penetration rate of 92% and 89.5% respectively in 2022 [31].

As a small and densely populated nation, Singapore was one of the first countries outside of China to identify COVID-19 cases and impose movement restrictions, leading to an increase in social media use [32] and the spread of several myths. For instance, some Singaporeans purchased Ivermectin for self-medication against COVID-19 [33], causing the government to publish advisories against using it [34].

Various fact-checking and myth-debunking platforms have emerged in response to the rise of misinformation, including the WHO’s Mythbusters [35]. In Singapore, an anti-fake news law passed in 2019 (Protection from Online Falsehoods and Manipulation Act, 2019) has been invoked to correct COVID-19 falsehoods, such as claims that the Omicron variant is resistant to vaccines [36]. Therefore, it is important to examine the changes in myth beliefs over time, to understand whether such refutation efforts have been successful.

The current study can inform future health communication efforts to tailor correction strategies to specific population subsets and more effectively prevent misinformation spread. They can also allow policy makers to understand if refutational messaging is effectively correcting beliefs or whether new methods are necessary to mitigate their impact.

## Method

### Data collection

Data were collected from two online surveys of nationally representative and cognitively intact adults, defined by our university’s ethics board as aged between 21 and 70 years, residing in Singapore. Ethics approval for the surveys were obtained from the Nanyang Technological University Institutional Review Board (approval numbers IRB-2020-08-014 and IRB-2021-1034). A target sample size of approximately 1200 participants for each survey was decided based on similar survey studies [23, 24] and to account for dropouts and incomplete responses. Participants were recruited through an expert online panel provider using randomised sampling and were invited via email to participate in a 20-minute online survey, with the option to leave at any point. After reading a detailed information sheet about the study, participants had to provide their written consent to participate before being allowed to proceed with the survey. Recruitment and administration of the first survey was conducted from 19 October 2020 to 24 February 2021. To examine changes in the prevalence of myths over time, a follow-up survey was conducted. Using a similar sample profile from the same panel provider, a separate set of participants was recruited using the same methods for this follow-up survey from 15 March 2022 to 6 April 2022. Both surveys were conducted in English, the most widely spoken language in Singapore, so as to ensure that participants were able to answer the questions comfortably. Each of the participants received an honorarium as a token of appreciation.

### Measures

Demographic information such as age, ethnicity, gender and education were collected in both surveys.

COVID-19 misinformation beliefs were selected through discussions with health communication experts and a review of the most widely reported myths in academic literature and Singapore’s mainstream news media (see Table 1). Across both surveys, seven highly popular false beliefs were examined. In the second survey administered in 2022, three additional myths were added based on the development of the pandemic. Participants responded to these myths with one of three options: “true”, “false” or “do not know”. As all the myths are incorrect, respondents were marked as correct only if they answered “false”, and incorrect if they selected “true” or “do not know”.

**Table 1:** Highly popular Singapore COVID-19 myths examined.

Myth	Possible basis for myth	Refutation
COVID-19 can be treated with antibiotics.	This myth could be a result of similar long-standing misconceptions within the infectious diseases space. Such false beliefs might have emerged because viral infections can sometimes lead to secondary infections that require antibiotics [37].	It is an established fact that antibiotics are only effective against bacterial infections and not viral ones. This myth has been debunked by several public health bodies, including the WHO [35].
You need to be with an infected person for 10 minutes to contract the virus.	Early data from China suggested that infections occurred with large amounts of close contact with an infected person [38]. In late 2020, the White House also reported that they would only trace the contacts of those who had spent over 15 minutes in close proximity to the COVID-19-positive president [39]. This led to false claims that COVID-19 transmission requires spending a significant amount of time with an infected person.	This has been debunked, with cases of minimal contact leading to COVID-19 infections [40].
Being able to hold my breath for 10 seconds or more without coughing or feeling discomfort means I don’t have COVID-19.	A common symptom of COVID-19 is a dry cough and those with severe disease can develop pneumonia. This might have been the basis of unfounded social media claims that breath-holding ability is an indicator of COVID-19 infection [41].	This claim is false, and it has been debunked by the WHO and public health experts [42].
If I wear a mask, I cannot get COVID-19.	Mask-wearing became mandatory in many countries during the first two years of the pandemic, which could have led people to believe it completely prevents one from getting infected.	Although wearing a mask can reduce the spread of the disease, it cannot guarantee protection against COVID-19.
COVID-19 is manmade and was released deliberately.	The relatively unknown origins of the virus early in the pandemic, and Donald Trump’s claim that COVID-19 was made in a laboratory in China [43] fuelled the spread of this myth.	Existing evidence points to a natural origin for the virus, with widespread consensus among the scientific community [44].
Eating garlic can prevent COVID-19.	Garlic has antimicrobial properties [45] that can be beneficial to health, which might have led to claims that it can prevent COVID-19 infection.	There is no evidence that garlic can be used preventatively against COVID-19 [46].
Exposing myself to sunlight above 25 degrees can prevent COVID-19.	Existing evidence indicates that viruses do not respond well to heat [47]. This might have led to the assumption that exposure to sunlight can kill the virus that causes COVID-19. In addition, vitamin D produced from sunlight has garnered interest as a preventive agent against COVID-19 [48].	This claim has been refuted by public health institutions and fact-checkers [49]. Countries with hot weather have also experienced high COVID-19 case numbers.
Ivermectin is safe and effective in treating COVID-19.	Early in 2020, some studies indicated that Ivermectin could prevent the virus from replicating [50]. Several Latin American countries also included the drug in their treatment guidelines [51]. In Singapore, an online petition called for Ivermectin to be used as an outpatient drug against COVID-19 [52].	Many public health bodies, including Singapore’s Health Sciences Authority have warned against using Ivermectin, as poisoning cases have been reported [53]. Studies that claimed that Ivermectin is effective have also been found to be flawed [54].
Child immune systems cannot handle so many vaccines (i.e. COVID-19 vaccine and other childhood vaccines against diseases such as influenza, MMR).	Prior to the pandemic, vaccine hesitancy was already considered one of the top ten public health threats [55]. Since COVID-19 vaccines became available, false claims about vaccination have gained traction [56].	This has been refuted by multiple health authorities around the world, as existing evidence does not indicate safety issues regarding the administration of several vaccines at the same time [57].
5G causes COVID-19.	False reports that 5G technology was first rolled out in Wuhan right before the pandemic began [58] and longstanding conspiracy theories about wireless technology [59] fuelled this myth.	There is no evidence of health risks associated with 5G technology [60].

### Data analysis

Data were analysed using IBM SPSS version 29 [61] and Stata version 15.1 [62]. Duplicate responses, those with missing data, and those who completed the survey in less than half of the median speed (suggesting they did not read the questions), were removed. Participants who exhibited extensive straight-lining behaviour (i.e., giving identical answers to multiple consecutive items, which suggests they were not providing genuine responses), and ineligible participants (i.e., those that were not residents of Singapore, outside of the age range or submitted responses from outside of Singapore) were also removed. Thus, a total of 1221 respondents participated in the first survey, of which 272 were removed. For the second survey in 2022, 1121 participants responded, of which 37 were removed.

To understand the changes in myth beliefs over time, the differences in the prevalence of the seven myths utilised in both the 2020 and 2022 survey questionnaires were assessed using crosstabulation and Pearson Chi-squared tests of independence. The relationship between demographic groups (gender, age groups, education and ethnicity) and all 10 myths were also examined using crosstabulation and Pearson Chi-squared tests of independence. For all analyses, a p-value of less than 0.05 was considered statistically significant.

## Results

Overall, 2,033 participants were included in this study, whose characteristics are summarised in Table 2. The 2020 survey was completed by 949 participants (M = 45.2 years, SD = 13.5 years; 457 males), while 1084 participants completed the follow-up 2022 survey (M = 41.8 years, SD = 12.9 years; 523 males). Overall, the samples for both years were similar in their demographic profiles, although the follow-up 2022 sample was slightly younger than the 2020 sample (*p* < 0.001). Both samples were generally representative of the resident Singapore population (comprising Singapore Citizens and Permanent Residents), although there was a higher proportion of those who received a university education and lower proportion of individuals who attained below secondary education level [28].

**Table 2:** Demographic profile of the study participants (N = 2033).

Demographic characteristic	Frequency (%)
Age in years (mean ± SD)	43.4 ± 13.3
Young adults (21–35)	686 (33.7)
Middle-aged adults (36–50)	683 (33.6)
Older adults (51–70)	664 (32.7)
Gender	
Male	980 (48.2)
Female	1053 (51.8)
Ethnicity	
Chinese	1581 (77.8)
Malay	239 (11.8)
Indian	142 (7.0)
Other	71 (3.5)
Highest education level	
No formal schooling	8 (0.4)
Primary level	27 (1.3)
Secondary and post-secondary level	942 (46.3)
Bachelor’s degree or higher	1056 (51.9)

### Belief in myths (RQ1)

Overall, examining data from both years combined (N = 2033), the proportion of participants who were unable to identify the myths ranged from 12.7% to 57.5%.

For the myth that “COVID-19 can be treated with antibiotics”, 63.7% correctly answered that this was false. Almost half of the participants could not recognise that it is a myth that one must spend at least 10 minutes with an infected person to get COVID-19 (48.0%).

Most participants were correctly aware that being able to hold their breath for at least 10 seconds was not an indicator of being uninfected (73.6%). The majority of people correctly believed that wearing a mask could not guarantee prevention from COVID-19 (78.4%). In contrast, over half the participants incorrectly believed or were unsure whether COVID-19 was manmade (57.5%). The majority of participants correctly answered that eating garlic and exposure to sunlight cannot prevent COVID-19.

For the three myths examined only in 2022, almost half of the participants were unsure or incorrectly believed that Ivermectin can treat COVID-19 (48.0%). Over half of the participants could not identify the myth that child immune systems cannot handle so many vaccines (51.5%), while 76.2% of participants correctly answered that 5G does not cause COVID-19.

### Changes in myth prevalence over time (RQ2)

**Fig 1:**
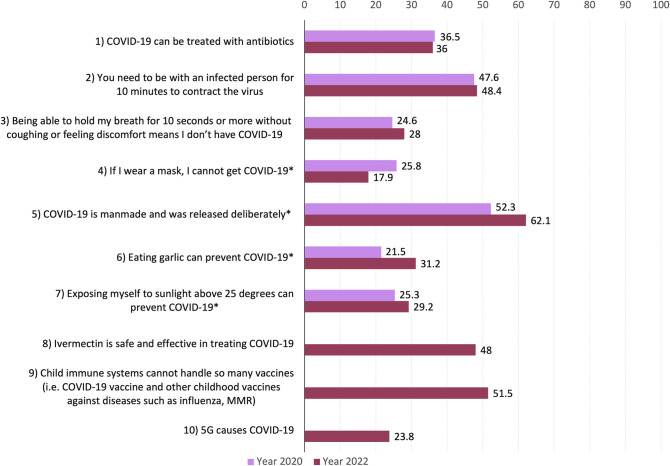
Percentage of incorrectly answered myths in 2020 versus 2022. Asterisk (*) indicates that there was a significant difference in the percentage that did not correctly answer this myth between the 2020 and 2022 survey. Statements 8 to 10 were only included in 2022. Answers were marked as incorrect if participants selected “true” or “do not know”.

Examining changes in myth prevalence over time, belief in several myths significantly changed from 2020 to 2022 (Fig 1). The results of Chi-squared tests evaluating how beliefs in the 7 myths (examined in both surveys) varied by year are summarised in Table 3. The proportion of people correctly believing that wearing a mask could not guarantee prevention from COVID-19 increased significantly (χ^2^ = 18.65, *p* < 0.001) by 7.9% between 2020 and 2022, from 74.2% to 82.1%. In contrast, the proportion of people incorrectly believing or not knowing whether COVID-19 was manmade increased significantly by 9.8% from 2020 to 2022 (χ^2^ = 20.19, *p* < 0.001). In 2022, a significantly higher proportion of participants (χ^2^ = 24.40, *p* < 0.001) either answered incorrectly or were unsure about whether eating garlic can prevent COVID-19 (31.2%) as compared to 2020 (21.5%). Similarly, the percentage of people that either answered incorrectly or were unsure about the myth that exposure to sunlight can prevent COVID-19 significantly increased from 25.3% in 2020 to 29.2% in 2022 (χ^2^ = 3.98, *p* = 0.046).

**Table 3:** Results of Chi-squared tests to examine differences in 7 myth beliefs between 2020 and 2022.

Myth statements	Number that incorrectly answered myth in 2020 (%)	Number that incorrectly answered myth in 2022 (%)	χ2	p-value
COVID-19 can be treated with antibiotics.	346 (36.5)	390 (36.0)	0.04	0.846
You need to be with an infected person for 10 minutes to contract the virus.	452 (47.6)	524 (48.4)	0.12	0.734
Being able to hold my breath for 10 seconds or more without coughing or feeling discomfort means I don’t have COVID-19.	233 (24.6)	303 (28.0)	3.01	0.083
If I wear a mask, I cannot get COVID-19.	245 (25.8)	194 (17.9)	18.65	< 0.001
COVID-19 is manmade and was released deliberately.	496 (52.3)	673 (62.1)	20.19	< 0.001
Eating garlic can prevent COVID-19.	204 (21.5)	338 (31.2)	24.40	< 0.001
Exposing myself to sunlight above 25 degrees can prevent COVID-19.	240 (25.3)	317 (29.2)	3.98	0.046

There were no significant differences between 2020 and 2022 for the myths that COVID-19 can be treated with antibiotics, one must spend at least 10 minutes with an infected person to get COVID-19, or that being able to hold one’s breath for at least 10 seconds was an indicator of being uninfected.

### Demographic factors and myth beliefs (RQ3)

**Fig 2:**
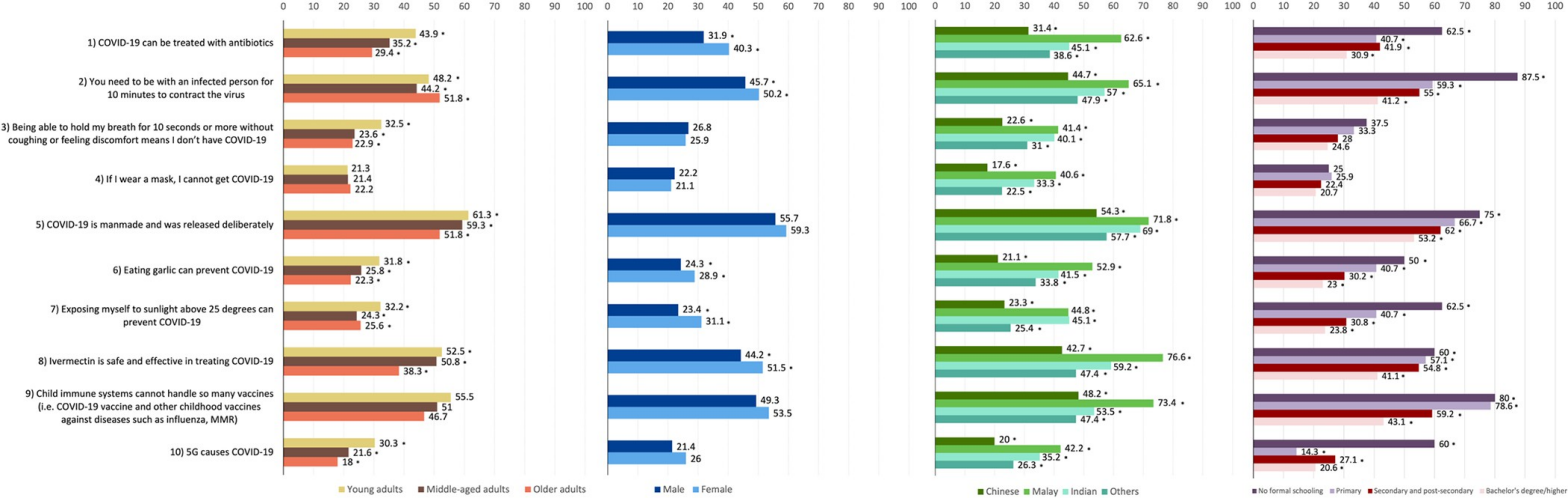
Percentage of participants within demographic groups who could not identify each myth as false. Asterisk (*) indicates that there were significant associations between the demographic variable and the myth.

In Chi-squared tests, combined survey data (N = 2033) was used to examine myths by age groups, gender, ethnicity and education level for the first seven myths, while 2022 data (N = 1084) was used for the last three myths that were only included in the second survey (see Fig 2 and Table 4). Results showed that a significantly lower proportion of females could correctly answer that “COVID-19 can be treated with antibiotics” is false (59.7%), compared to males (68.1%) (χ^2^ = 15.61, *p* < 0.001). There were significant differences between ethnicities for this myth belief (χ^2^ = 92.77, *p* < 0.001), with Chinese participants being the most likely to answer correctly that this myth is false (68.6%). Across all age groups, the majority of respondents were able to answer correctly, with 29.4% of 51–70-year-olds, 35.2% of 36–50-year-olds and 43.9% of 21–35-year-olds either answering incorrectly or being unsure about this myth (χ^2^ = 31.48, *p* < 0.001). Those who received a higher level of education were better at recognising this myth (χ^2^ = 28.55, *p* < 0.001).

**Table 4:** Results of Chi-squared tests to examine differences in proportion of participants who could not identify each myth as false, among demographic groups.

	Age groups				Gender			Ethnicity					Highest education level				
Myth statements	Young adults (%)	Middle-aged adults (%)	Older adults (%)	χ2 (*p*)	Male (%)	Female (%)	χ2 (*p*)	Chinese (%)	Malay (%)	Indian (%)	Other (%)	χ2 (*p*)	No formal schooling (%)	Primary level (%)	Secondary and post-secondary level (%)	Bachelor’s degree or higher (%)	χ2 (*p*)
COVID-19 can be treated with antibiotics.	301 (43.9)	240 (35.2)	195 (29.4)	31.48 (<0.001)	312 (31.9)	424 (40.3)	15.61 (<0.001)	496 (31.4)	149 (62.6)	64 (45.1)	27 (38.6)	92.77 (<0.001)	5 (62.5)	11 (40.7)	394 (41.9)	326 (30.9)	28.55 (<0.001)
You need to be with an infected person for 10 minutes to contract the virus.	330 (48.2)	302 (44.2)	344 (51.8)	7.78 (0.020)	447 (45.7)	529 (50.2)	4.26 (0.039)	706 (44.7)	155 (65.1)	81 (57.0)	34 (47.9)	39.70 (<0.001)	7 (87.5)	16 (59.3)	518 (55.0)	435 (41.2)	44.17 (<0.001)
Being able to hold my breath for 10 seconds or more without coughing or feeling discomfort means I don’t have COVID-19.	223 (32.5)	161 (23.6)	152 (22.9)	20.20 (<0.001)	263 (26.8)	273 (25.9)	0.22 (0.641)	358 (22.6)	99 (41.4)	57 (40.1)	22 (31.0)	53.85 (<0.001)	3 (37.5)	9 (33.3)	264 (28.0)	260 (24.6)	4.18 (0.243)
If I wear a mask, I cannot get COVID-19.	146 (21.3)	146 (21.4)	147 (22.2)	0.189 (0.910)	217 (22.2)	222 (21.1)	0.35 (0.553)	279 (17.6)	97 (40.6)	47 (33.3)	16 (22.5)	76.95 (<0.001)	2 (25.0)	7 (25.9)	211 (22.4)	219 (20.7)	1.19 (0.755)
COVID-19 is manmade and was released deliberately.	420 (61.3)	405 (59.3)	344 (51.8)	13.79 (0.001)	545 (55.7)	624 (59.3)	2.68 (0.102)	859 (54.3)	171 (71.8)	98 (69.0)	41 (57.7)	34.25 (<0.001)	6 (75.0)	18 (66.7)	584 (62.0)	561 (53.2)	17.80 (<0.001)
Eating garlic can prevent COVID-19.	218 (31.8)	176 (25.8)	148 (22.3)	15.93 (<0.001)	238 (24.3)	304 (28.9)	5.39 (0.020)	333 (21.1)	126 (52.9)	59 (41.5)	24 (33.8)	127.32 (<0.001)	4 (50.0)	11 (40.7)	284 (30.2)	243 (23.0)	18.12 (<0.001)
Exposing myself to sunlight above 25 degrees can prevent COVID-19.	221 (32.2)	166 (24.3)	170 (25.6)	12.37 (0.002)	229 (23.4)	328 (31.1)	15.45 (<0.001)	368 (23.3)	107 (44.8)	64 (45.1)	18 (25.4)	72.21 (<0.001)	5 (62.5)	11 (40.7)	290 (30.8)	251 (23.8)	19.80 (<0.001)
Ivermectin is safe and effective in treating COVID-19.	210 (52.5)	195 (50.8)	115 (38.3)	15.67 (<0.001)	231 (44.2)	289 (51.5)	5.85 (0.016)	362 (42.7)	98 (76.6)	42 (59.2)	18 (47.4)	54.78 (<0.001)	3 (60.0)	8 (57.1)	285 (54.8)	224 (41.1)	20.81 (<0.001)
Child immune systems cannot handle so many vaccines.	222 (55.5)	196 (51.0)	140 (46.7)	5.40 (0.067)	258 (49.3)	300 (53.5)	1.86 (0.172)	408 (48.2)	94 (73.4)	38 (53.5)	18 (47.4)	28.80 (<0.001)	4 (80.0)	11 (78.6)	308 (59.2)	235 (43.1)	33.50 (<0.001)
5G causes COVID-19.	121 (30.3)	83 (21.6)	54 (18.0)	15.75 (<0.001)	112 (21.4)	146 (26.0)	3.17 (0.075)	169 (20.0)	54 (42.2)	25 (35.2)	10 (26.3)	36.01 (<0.001)	3 (60.0)	2 (14.3)	141 (27.1)	112 (20.6)	10.64 (0.014)

Significant differences in ethnicity were also found for the myth that one needs to be with an infected person for 10 minutes to contract the virus (χ^2^ = 39.70, *p* < 0.001), with 44.7% of Chinese and 47.9% of Other ethnicities answering incorrectly or being unsure about this myth, as compared to 57% of Indians and 65.1% of Malays. There were also significant differences between genders (χ^2^ = 4.26, *p* = 0.039), with 50.2% of females more likely to incorrectly answer or select the “do not know” option, as compared to 45.7% of males. There were significant differences by age (χ^2^ = 7.78, *p* = 0.020), with 48.2% of 21–35-year-olds, 44.2% of 36–50-year-olds, and 51.8% of 51–70-year-olds answering incorrectly. Participants who were more highly educated were better able to identify this myth (χ^2^ = 44.17, *p* < 0.001).

22.6% of Chinese and 31% of Other ethnicities answered incorrectly or were unsure about the myth that being able to hold one’s breath for 10 seconds or more is an indicator that a person does not have COVID-19, in comparison to 41.4% of Malays and 40.1% of Indians (χ^2^ = 53.85, *p* < 0.001). Although the majority of participants in all three age groups answered correctly, those aged 21 to 35 (32.5%) and 36 to 50 (23.6%) were significantly more likely to answer incorrectly or be unsure about this myth, compared to those aged 51 to 70 (22.9%) (χ^2^ = 20.20, *p* < 0.001). There were no significant differences in gender or education level.

For the least prevalent myth that wearing a mask ensures one will not get COVID-19, Malays (40.6%) and Indians (33.3%) were more likely to answer incorrectly or be unsure about this myth, in comparison to those of Chinese (17.6%) or Other (22.5%) ethnicities (χ^2^ = 76.95, *p* < 0.001). No significant differences were found for gender, age groups and education, with the majority of participants across these demographic groups answering correctly.

Examining the most pervasive myth that COVID-19 is manmade and was released deliberately, 51.8% of older participants incorrectly responded or were unsure whether this was true, in comparison to 59.3% within the middle-aged group and 61.3% in the younger group (χ^2^ = 13.79, *p* = 0.001). Malays (71.8%) and Indians (69.0%) were significantly more likely to incorrectly believe or be unsure about whether COVID-19 was manmade than Chinese (54.3%) and Other ethnicities (57.7%) (χ^2^ = 34.25, *p* < 0.001). Those with lower education levels were less able to identify this myth as false (χ^2^ = 17.80, *p* < 0.001). There were no significant differences between genders.

For the myth that eating garlic can prevent COVID-19, significant differences were found within all four demographic variables. 28.9% of females were unable to identify this myth, in comparison to 24.3% of males (χ^2^ = 5.39, *p* = 0.020). Malays were the only ethnic group where a greater proportion (52.9%) answered incorrectly or were unsure (χ^2^ = 127.32, *p* < 0.001). While most participants in all age groups correctly identified that the statement is a myth, 51 to 70-year-olds were the most likely to answer correctly (77.7%) (χ^2^ = 15.93, *p* < 0.001). Those with bachelor’s degrees or higher qualifications were the least likely (23%) to answer incorrectly or be unsure about this myth (χ^2^ = 18.12, *p* < 0.001).

The majority of males and females answered correctly that exposing oneself to sunlight above 25 degrees cannot prevent COVID-19. However, females were significantly less likely to answer correctly (68.9%) as compared to males (76.6%) (χ^2^ = 15.45, *p* < 0.001). Most respondents in all ethnic groups could correctly identify this statement as a myth, but Malays (55.2%) and Indians (54.9%) were significantly less able to identify this myth compared to Chinese (76.7%) and Other ethnicities (74.6%) (χ^2^ = 72.21, *p* < 0.001). While most participants in all age groups did not believe this myth, those 36 to 50 years old were the least likely to incorrectly believe or be unsure about this myth (24.3%) (χ^2^ = 12.37, *p* = 0.002). Those with no formal schooling were the most likely to answer incorrectly or be unsure about this myth compared to those with higher education levels (χ^2^ = 19.80, *p* < 0.001).

Examining ethnicity, 42.7% of Chinese and 47.4% of Other ethnicities answered incorrectly or were unsure about the myth that Ivermectin is safe and effective in treating COVID-19, and this proportion was significantly higher in Indians (59.2%) and highest in Malays (76.6%) (χ^2^ = 54.78, *p* < 0.001). There were significant differences in gender (χ^2^ = 5.85, *p* = 0.016), with 55.8% of males correctly identifying this myth as false, in comparison to 48.5% of females. In terms of age, younger participants were more likely to answer incorrectly or respond with “do not know” for this myth (52.5%) than those aged 36 to 50 (50.8%) and 51 to 70 (38.3%) (χ^2^ = 15.67, *p* < 0.001). Significant differences among those of different education levels were also observed, where bachelor’s and postgraduate degree holders combined were the only group where a majority of respondents could identify this myth (χ^2^ = 20.81, *p* < 0.001).

Similarly, Chinese and Other ethnicities were significantly less likely to be incorrect or unsure about the myth that child immune systems cannot handle so many vaccines (48.2% and 47.4%, respectively), in comparison to Malays (73.4%) and Indians (53.5%) (χ^2^ = 28.80, *p* < 0.001). The pattern of differences in education levels was also similar to the previous myths discussed, with only 20% of those with no formal schooling able to identify this myth, in comparison to 56.9% of those with at least a bachelor’s degree (χ^2^ = 33.50, *p* < 0.001). There were no significant differences between genders and age groups.

Concerning the myth that 5G causes COVID-19, Malays (57.8%) and Indians (64.8%) were less able to correctly identify this myth, compared to Chinese (80%) and Other (73.7%) ethnicities (χ^2^ = 36.01, *p* < 0.001). While the majority of participants across age groups answered correctly, those aged 51 to 70 showed the best performance (82%) (χ^2^ = 15.75, *p* < 0.001). There were no significant differences between genders. Finally, while a higher proportion (85.7%) of those who received primary education correctly identified this myth in comparison to other education levels, the overall pattern of results showed that those with higher education levels generally performed better (χ^2^ = 10.64, *p* = 0.014).

## Discussion

In times of crisis, falsehoods are quick to spread, causing erroneous beliefs and unwanted behaviours that can be detrimental to society. This is evinced in the case of the COVID-19 pandemic, which has spurred a surge of misinformation worldwide. The current study examined the level of belief in ten popular myths within Singapore over two timepoints during the pandemic and how these beliefs varied by demographic profiles. Identifying the most prevalent myths and the types of people most vulnerable to them can aid health policymakers in designing targeted health communication strategies to dispel not only COVID-19 myths, but also counter other myths during infectious diseases outbreaks and future health crises.

### Beliefs in individual myths

The first objective of this study was to understand the strength of belief in prevalent COVID-19 myths in a multicultural Asian setting. Our findings showed that the number of people who could not identify the myths as false varied. For the most widely held myth that COVID-19 was manmade and released deliberately, over half of the participants were unable to identify this myth as false at both timepoints. This could be due to the ongoing debate about the virus’ origins during the periods where the surveys were administered, which has been reinvigorated several times with new findings reported by respectable scientists and news outlets [63]. With novel and contradicting ideas about COVID-19’s origins spreading, laypersons may have thought that there was no consensus on this matter. The results mirror a study in Jordan where close to 60% of respondents believed COVID-19 is a man-made disease [64].

The second most prevalent myth was that child immune systems cannot handle so many vaccines (i.e. COVID-19 vaccine and other routine childhood vaccinations). This is concerning given the considerable parental hesitancy around child COVID-19 vaccination in Singapore (15.9% compared to 9.9% hesitancy for adult own vaccination) [65] and lowered uptake of vaccines for measles [66]. Parents are more risk-averse when vaccinating younger children [67] and may be especially attuned to concerns about safety and long-term effects. This follows other prominent falsehoods about children’s vaccines in recent years, leading to a decline in childhood vaccinations [68]. These findings underscore the need to bolster existing efforts to address parental hesitancy towards child vaccination, in preparation for future health crises.

The high level of beliefs in myths can be concerning, particularly if the myths lead to harmful behaviours. Over a third of participants incorrectly believed or were unsure whether antibiotics can be used to treat COVID-19, despite local awareness campaigns to deter antibiotic misuse [69]. Overuse of antibiotics is problematic as it can lead to the spread of antimicrobial resistance [70]. Similarly, almost half of the participants believed or were not sure if one needed to be in contact with an infected person for 10 minutes to get COVID-19, even though this has been debunked [39]. Therefore, health communicators need to find novel approaches to promote the correct preventative behaviours during infectious disease outbreaks.

Some myths, such as 5G causes COVID-19 and people wearing masks cannot get COVID-19 had low levels of incorrect beliefs. This contrasts with Western countries, such as the United States, where mask-wearing has been a controversial issue [71], or the United Kingdom, where 5G towers have been attacked after the spread of 5G myths [20]. This could be because mask-wearing was mandatory and highly regulated in Singapore from April 2020 to August 2022 [72, 73], driving adherence to this behaviour [74]. Despite this, Singapore still experienced multiple waves of COVID-19 cases, highlighting that mask-wearing alone cannot eliminate infection. In contrast, in countries like the United States, mask wearing requirements and adherence varied across different states at various times [75]. Additionally, Singapore is ranked as one of the top smart cities in the world [30] and is highly digitally connected, hence, many residents are less wary of technology in their lives, which makes it less likely that myths about 5G can propagate.

### Changes in myth beliefs over time

The second objective of this study was to examine how beliefs in COVID-19 myths have changed over time. We found that many myths remained prevalent over the pandemic, either remaining at similar levels or increasing, despite public debunking from the government and national newspapers. This could be due to the uncertainty surrounding the disease, which can result in stress, fear and a loss of control over one’s health [76]. Such feelings can deepen conspiracy beliefs [77, 78]. Furthermore, anger became a prominent emotion as the pandemic progressed [79], which can fuel the spread of misinformation [80]. In tandem with increased information-seeking behaviours to make sense of the crisis [81], this could have led to the rapid circulation of misinformation and greater exposure to myths, which in turn is associated with higher belief in misinformation [18]. Future research should continue to examine the factors that allow myth beliefs to persist. Effective communication strategies should also account for the uncertainties and emotional responses that people are experiencing in times of crisis, to help to foster a sense of control.

The fact that these myths remained prevalent is of particular interest as research has found a strong link between higher trust in government and lower belief in false information [82]. Several studies have found that Singaporeans have a high level of trust in authorities regarding COVID-19 information [83], which should have led to lower myth beliefs among the population. Hence, trust should be studied in relation to myths in the future to better understand how they are linked in the Singapore population.

### Demographic factors and myth beliefs

Finally, the last aim of this study was to examine the demographic factors associated with myth beliefs. The results indicate that inequities in misinformation beliefs and knowledge regarding COVID-19 exist, even in a highly educated country with a high Internet penetration rate [84]. Across the 10 myths examined, females, ethnic minorities (Malays and Indians) and those from younger age groups were more vulnerable to misinformation.

Examining myths by gender, males are better able to correctly identify the myths as incorrect compared to females. This is consistent with existing research demonstrating that women are more likely to believe COVID-19 misinformation [82] and more likely to accept general health misinformation [85]. However, other studies have shown that males demonstrate higher beliefs in COVID-19 misinformation [86]. These contradictory findings should be further investigated to identify why gender differences exist.

More attention needs to be paid to debunking myths for those in the younger age groups of 21 to 35 in the future, as they were more likely to believe in or be unsure about the myths compared to other age groups. Similar findings were also reported in multi-country studies on coronavirus misinformation [24, 86] and health misinformation [85]. Additionally, younger adults in Singapore are more likely to use social media [87]. Frequent social media use was associated with greater beliefs in particular types of COVID-19 misinformation in the United States [88]. Indeed, De Coninck and colleagues’ [89] study found that lower misinformation and conspiracy beliefs were observed among those who frequently used traditional media sources. In contrast, greater information-seeking from online platforms was associated with greater misinformation beliefs.

Chinese respondents, who are the majority ethnic group within Singapore, were the least likely to believe in the myths out of all the ethnic groups. This echoes recent research where those who identified as a minority group member were more susceptible to COVID-19 misinformation [24]. Respondents who belong to the ethnic group categorised as ‘Other’ were the second least likely to believe in myths after the Chinese respondents. This pattern of results could be because both groups largely consist of highly educated people [28], and higher education levels are associated with lower sharing and believing of COVID-19 rumours [90].

The Indian population within Singapore were the most likely to believe in or be unsure about the myths, followed by the Malay ethnic group. This could be due to both ethnic groups having higher proportions of people who ascribe to a particular faith, in comparison to the Chinese and Other populations [28], as research has found that higher religiosity is associated with greater belief in conspiracy theories [91]. In addition, Al-Zaman [92] found that out of 138 countries, India was the most affected by misinformation and produced the most misinformation. Many Singaporean residents of Indian ethnicity have extended family residing in India and thus may have been more exposed to myths than the other ethnicities. Effective health communication strategies in future health crises will need to focus on these vulnerable population sub-groups.

Finally, those with lower education levels were less likely to correctly identify the myths. Similar disparities in myth beliefs have been found across multiple existing studies, which indicate that higher education levels are negatively associated with belief in Covid-19 misinformation and conspiracy theories [82, 85, 86, 89, 90]. This highlights a possible gap in public health messaging for those with lower educational attainment.

### Limitations and future directions

This study has a few limitations. Firstly, although this was one of the first studies in Southeast Asia to examine myths over time, different cohorts (with similar demographic profiles) were used between the first and second surveys. Hence, we could not compare direct changes in the myth beliefs of the same cohort over time. Secondly, the surveys were conducted online, which might have skewed the data towards more proficient Internet users. Additionally, the surveys were conducted in English, which might exclude non-English-speaking respondents from participation. However, English is the most widely used language in Singapore [29], and the samples for both surveys were representative of the national adult population.

Future studies should examine the attitudes and media use behaviours of the different demographic groups, to better understand the pattern of associations between media utilisation and myth beliefs. Further, comparisons with myth beliefs in other countries can be investigated to see if there are cross-cultural and regional patterns of relationships. Finally, to obtain a more comprehensive understanding of trends in myth beliefs over time in health crises, surveys can be conducted at timepoints where significant events occurred, such as the peak of cases or introduction of key measures.

### Implications and conclusion

To our knowledge, this is one of the first studies examining the levels of belief in a range of COVID-19 myths over time in Southeast Asia. The findings have practical implications for health communication efforts to reduce misinformation beliefs during crisis situations. The data indicate that, despite debunking efforts, the prevalence of myths remained high at two time points in the pandemic. This could lead to detrimental consequences, such as the perpetuation of dangerous health behaviours and the further spread of falsehoods. Beyond misinformation correction, other health messaging strategies are needed to manage long-standing myths in future health crises. The major dissimilarities observed across population sub-groups also suggest that efforts focussed on vulnerable population segments will be necessary to counter these erroneous beliefs. Policymakers should pursue a more targeted health communication approach that engages younger populations, females, ethnic minorities and those with lower education levels to debunk and reduce the spread of health myths. Our findings also point to the need for myths to be addressed longitudinally during crises, instead of through one-time mass campaign approaches.

## Supporting information

S1 Table Results of binary logistic regression analyses to examine differences in proportion of participants who could not identify each myth as false, among demographic groups. (DOCX)

S1 Dataset Minimal anonymized dataset containing survey data for 2020 and 2022. (SAV)
