# Supplementary material for: You must be myths-taken: Examining belief in falsehoods during the COVID-19 health crisis
Source: PLoS One. 2024 Mar 5;19(3):e0294471. doi: 10.1371/journal.pone.0294471 (PMC10914263; doi:10.1371/journal.pone.0294471)
Supplement: S1 Table — (DOCX) [file pone.0294471.s001.docx]

| **Myth statements** | **Age groups (OR, 95% CI, *p*)** | | | **Gender (OR, 95% CI, *p*)** | | **Ethnicity (OR, 95% CI, *p*)** | | | | **Highest education level (OR, 95% CI, *p)*** | | | |
| --- | --- | --- | --- | --- | --- | --- | --- | --- | --- | --- | --- | --- | --- |
|  | Young adults | Middle-aged adults | Older adults | Male | Female | Chinese | Malay | Indian | Other | No formal schooling | Primary level | Secondary and post-secondary level | Bachelor's degree or higher |
| COVID-19 can be treated with antibiotics. | (ref) | 1.44  [1.16, 1.80]  < 0.001 | 1.89  [1.51, 2.36]  < 0.001 | (ref) | 0.69  [0.58, 0.83]  < 0.001 | (ref) | 0.27  [0.21, 0.36]  < 0.001 | 0.56  [0.39, 0.79]  < 0.001 | 0.73  [0.45, 1.19]  0.207 | (ref) | 2.42  [0.48, 12.30]  0.285 | 2.31  [0.55, 9.74]  0.253 | 3.73  [0.89, 15.69]  0.073 |
| You need to be with an infected person for 10 minutes to contract the virus. | (ref) | 1.17  [0.95, 1.45]  0.142 | 0.87  [0.70, 1.07]  0.182 | (ref) | 0.83  [0.70, 0.99]  0.039 | (ref) | 0.43  [0.33, 0.57]  < 0.001 | 0.61  [0.43, 0.86]  0.005 | 0.88  [0.55, 1.41]  0.592 | (ref) | 4.81  [0.52, 44.82]  0.168 | 5.73  [0.70, 46.75]  0.103 | 9.98  [1.22, 81.38]  0.032 |
| Being able to hold my breath for 10 seconds or more without coughing or feeling discomfort means I don’t have COVID-19. | (ref) | 1.56  [1.23, 1.98]  < 0.001 | 1.62  [1.27, 2.07]  < 0.001 | (ref) | 1.05  [0.86, 1.28]  0.641 | (ref) | 0.41  [0.31, 0.55]  < 0.001 | 0.44  [0.31, 0.62]  < 0.001 | 0.65  [0.39, 1.10]  0.105 | (ref) | 1.20  [0.23, 6.19]  0.827 | 1.54  [0.37, 6.49]  0.556 | 1.84  [0.44, 7.74]  0.407 |
| If I wear a mask, I cannot get COVID-19. | (ref) | 0.99  [0.77, 1.29]  0.966 | 0.95  [0.73, 1.23]  0.692 | (ref) | 1.07  [0.86, 1.32]  0.553 | (ref) | 0.31  [0.24, 0.42]  < 0.001 | 0.43  [0.30, 0.62]  < 0.001 | 0.74  [0.42, 1.31]  0.294 | (ref) | 0.95  [0.16, 5.86]  0.958 | 1.15  [0.23, 5.76]  0.862 | 1.27  [0.26, 6.36]  0.768 |
| COVID-19 is manmade and was released deliberately. | (ref) | 1.09  [0.88, 1.35]  0.446 | 1.47  [1.19, 1.83]  < 0.001 | (ref) | 0.86  [0.72, 1.03]  0.102 | (ref) | 0.47  [0.35, 0.63]  < 0.001 | 0.53  [0.37, 0.77]  < 0.001 | 0.87  [0.54, 1.41]  0.572 | (ref) | 1.50  [0.25, 8.98]  0.657 | 1.84  [0.37, 9.16]  0.457 | 2.64  [0.53, 13.15]  0.235 |
| Eating garlic can prevent COVID-19. | (ref) | 1.34  [1.06, 1.69]  0.015 | 1.62  [1.27, 2.07]  < 0.001 | (ref) | 0.79  [0.65, 0.96]  0.020 | (ref) | 0.24  [0.18, 0.31]  < 0.001 | 0.38  [0.26, 0.54]  < 0.001 | 0.52  [0.32, 0.87]  0.012 | (ref) | 1.46  [0.30, 7.09]  0.643 | 2.31  [0.58, 9.32]  0.238 | 3.35  [0.83, 13.48]  0.089 |
| Exposing myself to sunlight above 25 degrees can prevent COVID-19. | (ref) | 1.48  [1.17, 1.88]  0.001 | 1.38  [1.09, 1.75]  0.008 | (ref) | 0.67  [0.55, 0.82]  < 0.001 | (ref) | 0.37  [0.28, 0.50]  < 0.001 | 0.37  [0.26, 0.53]  < 0.001 | 0.89  [0.52, 1.54]  0.686 | (ref) | 2.42  [0.48, 12.30]  0.285 | 3.75  [0.89, 15.78]  0.072 | 5.35  [1.27, 22.52]  0.022 |
| Ivermectin is safe and effective in treating COVID-19. | (ref) | 1.07  [0.81, 1.42]  0.630 | 1.78  [1.31, 2.41]  < 0.001 | (ref) | 0.75  [0.59, 0.95]  0.016 | (ref) | 0.23  [0.15, 0.35]  < 0.001 | 0.52  [0.32, 0.84]  0.008 | 0.83  [0.43, 1.59]  0.573 | (ref) | 1.13  [0.14, 9.00]  0.912 | 1.24  [0.21, 7.46]  0.817 | 2.15  [0.36, 12.97]  0.404 |
| Child immune systems cannot handle so many vaccines. | (ref) | 1.20  [0.90, 1.58]  0.211 | 1.43  [1.06, 1.93]  0.021 | (ref) | 0.85  [0.67, 1.08]  0.173 | (ref) | 0.34  [0.22, 0.51]  < 0.001 | 0.81  [0.50, 1.31]  0.387 | 1.03  [0.54, 2.00]  0.923 | (ref) | 1.09  [0.09, 13.78]  0.946 | 2.75  [0.31, 24.81]  0.367 | 5.28  [0.59, 47.52]  0.138 |
| 5G causes COVID-19. | (ref) | 1.57  [1.14, 2.17]  0.006 | 1.98  [1.37, 2.84]  < 0.001 | (ref) | 0.78  [0.59, 1.03]  0.075 | (ref) | 0.34  [0.23, 0.50]  < 0.001 | 0.46  [0.27, 0.77]  0.003 | 0.70  [0.33, 1.47]  0.342 | (ref) | 9.00  [0.87, 92.76]  0.065 | 4.03  [0.67, 24.38]  0.129 | 5.80  [0.96, 35.13]  0.056 |
